# Differentially expressed tRNA-derived fragments and their roles in primary cardiomyocytes stimulated by high glucose

**DOI:** 10.3389/fendo.2022.1049251

**Published:** 2023-01-13

**Authors:** Yongting Zhao, Ruxin Wang, Qi Qin, Jiaojiao Yu, Hui Che, Lihong Wang

**Affiliations:** ^1^ Department of Endocrinology, Second Affiliated Hospital of Harbin Medical University, Harbin, China; ^2^ Department of Endocrinology and Metabolism, First Affiliated Hospital of Jinan University, Guangzhou, China; ^3^ Innovation Center for Neurological Disorders, Department of Neurology, Xuanwu Hospital, Capital Medical University, Beijing, China

**Keywords:** tRNA-derived fragments, diabetic cardiomyopathy, cardiomyocyte injury, autophagy, ATG5

## Abstract

Diabetic cardiomyopathy (DCM) is a serious complication of diabetes mellitus that can cause malignant arrhythmia and sudden death and is associated with cardiomyocyte dysfunction induced by hyperglycemia. Emerging evidence has revealed that transfer RNA-derived fragments (tRFs), a novel class of noncoding RNAs, play a crucial role in a variety of pathophysiologic processes, including cell death, cell growth and proliferation. However, it remains unknown whether and how tRFs are involved in cardiomyocyte dysfunction during the progression of DCM. In this study, we found that cardiomyocyte abnormalities were induced by high glucose (HG) treatment, as demonstrated by a decrease in cell viability and autophagy activation as well as an increase in cell death and proinflammatory cytokine release. Moreover, HG treatment resulted in differential expression of tRFs in cardiomyocytes, of which 4 upregulated and 1 downregulated tRFs were observed compared with the control group. The differential expression of 4 upregulated tRFs was primarily involved in cardiac dysfunction-related processes, such as autophagy, AGE-RAGE signaling pathway in diabetic complications, MAPK signaling pathway, insulin signaling pathway, FoxO signaling pathway, insulin resistance and peroxisome pathways based on Kyoto Encyclopedia of Genes and Genomes (KEGG) pathway enrichment analysis. Furthermore, we found that tRF-5014a, the most significantly upregulated tRF among all tested tRFs, negatively regulated the expression of the autophagy-related protein ATG5. Importantly, inhibition of tRF-5014a not only abolished autophagy inactivation but also attenuated the decrease in cell viability and increase in cell death as well as proinflammatory cytokine release under HG conditions. These findings suggest that tRFs may contribute to HG-induced cardiomyocyte injury during DCM progression.

## 1 Introduction

Diabetic cardiomyopathy (DCM) is one of the important causes of heart failure, malignant arrhythmia and sudden death in patients with diabetes with a manifestation of abnormal myocardial structure and function (1, 2). There are multiple pathophysiological abnormalities in DCM, such as hyperglycemia, insulin resistance, oxidative stress and inflammation (3). Among them, cardiomyocyte dysfunction plays a significant role in the occurrence and development of DCM (4, 5). Despite measures to control blood sugar and improve heart function, the incidence rate and mortality rate of DCM remain high. Therefore, it is imperative to further explore its mechanism.

Transfer RNA-derived fragments (tRFs), a novel family of short noncoding RNAs with a length of less than 30 nt, are derived from precursor or mature tRNAs and are exceedingly present and conserved in most organisms (6–8). Emerging evidence suggests that tRFs play a crucial role in a variety of pathological and physiological processes, such as proliferation, cell growth, translation regulation, DNA damage and apoptosis escape (9–15). In addition, studies have shown that tRFs can act as biomarkers for a variety of tumors, such as breast, colorectal and prostate cancers (16–18). Some studies have revealed that tRFs exert translation silencing by serving as microRNAs (miRNAs), which bind with mRNAs and regulate their stability (19, 20). Moreover, tRFs can replace the 3’ untranslated region (UTR) from the RNA-binding protein YBX1, inhibiting the stability of manifold carcinogenic transcripts in breast carcinoma cells (21). Shao et al. reported that inhibition of tRF-Leu-CAG may inhibit cell proliferation and the cell cycle of non-small cell lung cancer (NSCLC) by inhibiting AURKA (22). Zhou et al. demonstrated that upregulation of tRF5-Glu can inhibit the proliferation of ovarian cancer cells by regulating BCAR3 expression (23). However, whether tRFs are associated with DCM is still largely unknown.

In the present study, we aimed to identify the expression and potential role of tRFs in primary cardiomyocytes stimulated with high glucose (HG). Our results suggested that HG caused cardiomyocyte injury accompanied by differentially expressed tRFs. Specifically, inhibition of tRF-5014a, the most significantly upregulated tRF, alleviated cardiomyocyte injury by regulating autophagy under HG conditions. These findings suggest that tRFs may contribute to HG-induced cardiomyocyte injury during the progression of DCM.

## 2 Materials and methods

### 2.1 Cell culture and treatment

Primary cardiomyocytes were extracted from 1- to 3-day-old neonatal mice and cultured in Dulbecco’s modified Eagle’s medium (DMEM) (HyClone, Logan, UT, USA) containing 10% fetal bovine serum (FBS) and 1% penicillin/streptomycin at 37°C with 95% humidity and 5% CO_2_. After 48 h, the culture medium was changed. The cells were used for experiments.

### 2.2 Cell transfection

High glucose (HG) treatment is a common method to simulate DCM *in vitro* (24). Cells were treated with low glucose (5.5 mM, control) and high glucose (50 mM, HG). At approximately 70% confluence, the media were replaced with culture media containing different glucose concentrations, and the cells were transiently transfected with tRF-5014a mimic (mimic-5014a), tRF-5014a inhibitor (inhibitor-5014a), tRF-5014a mimic negative control (mimic-NC) or tRF-5014a inhibitor negative control (inhibitor -NC) (RIBOBIO, Guangzhou, China) using X-treme GENE transfection reagent (Roche, Mannheim, Germany) according to the manufacturer’s protocol. Protein and RNA were collected after 72 hours.

### 2.3 tRF and tiRNA PCR array

In this study, a commercial tRF and tiRNA PCR array from ArrayStar was used to examine 88 tRFs known to be amplified by PCR. TRIzol reagent (Invitrogen, Carlsbad, CA, USA) was used to extract total RNA from the cells. The concentration of the RNA was measured using a NanoDrop ND-1000 (Thermo Fisher Scientific, USA). First, DNase treatment and RNA cleanup were performed using a RNeasy^®^ MinEluteTM cleanup kit (Qiagen, Germany). The rtStarTM tRF&tiRNA pretreatment kit (Cat# AS-FS-005, Arraystar, USA) and rtStarTM First-Strand cDNA Synthesis kit (Cat# AS-FS-003, Arraystar, USA) were applied separately to pretreat tRF and tiRNA and synthetize first-strand cDNA with tRF-specific RT primers. The process included the following steps: 3’-terminal deacylation, 3’-cP removal and 5’-P addition, demethylation, 3’ adaptor ligation, reverse transcription primer hybridization, 5’ adaptor ligation and reverse transcription. Arraystar SYBR^®^ Green qPCR Master Mix (ROX+) (AS-MR-006-5, Arraystar, USA) was used to perform qRT-PCR, amplifying and quantifying the cDNA according to the manufacturer’s protocols. The expression levels of tRFs were standardized to that of 5S RNA, and the relative expression levels of genes were quantified by the 2^-ΔΔCT^ method.

### 2.4 Western blot analysis

Total protein samples were extracted using RIPA lysis buffer; after adding loading buffer, the proteins were separated *via* 12.5% SDS-PAGE and transferred onto nitrocellulose membranes, which were subsequently blocked with blocking buffer. Afterward, the membranes were incubated with primary antibodies against ATG5 (1:1000, Abcam, ab108327, UK), LC3 (1:1000, Sigma, USA), and β-actin (1:1000, Bioss, China) at 4°C overnight. After that, the membranes were washed three times with PBS containing 0.5% Tween 20 (PBS-T) and incubated with the corresponding secondary antibodies for 1 h at room temperature. β-actin served as an internal control. Images were captured with GelDox XR System (Bio-Rad, CA, USA), and the densities of bands were quantified with a Quantity One system.

### 2.5 Quantitative real-time PCR

Reverse transcription was achieved using a reverse transcription kit (Toyobo, Japan) at 42°C for 60 min and then at 70°C for 10 min. The tRF-5014a-specific RT-primer, which was designed by RIBOBIO (Guangzhou, China), was used instead of random primer to detect the level of tRF-5014a. The tRF-5014a-specific forward primer was also designed by RIBOBIO (Guangzhou, China). An ABI 7500 Fast Real-Time PCR system (Applied Biosystems, CA, USA) was used to perform real-time PCR, amplifying and quantifying cDNA using SYBR Green I (Toyobo, Osaka, Japan) according to the manufacturer’s protocols. The expression levels of tRFs were standardized to U6. The relative expression levels of genes were quantified by the 2^-ΔΔCT^ method.

### 2.6 Immunofluorescence staining

To fix cells, 4% buffered paraformaldehyde was applied at room temperature, after which the cells were blocked for 2 h at room temperature with 1% BSA and 0.1% Triton-X, followed by incubation with primary antibody against ATG5 at 4°C overnight. The next day, the cells were treated with the corresponding secondary antibody for 1 h at room temperature. Nuclei were stained with DAPI (Beyotime, Shanghai, China) for 20 min. Images were captured by fluorescence microscopy and quantified *via* Image-Pro Plus 6.0.

### 2.7 Cell viability assay

Cell Counting Kit-8 (CCK-8) assays were conducted according to the manufacturer’s instructions to assess viability. Primary cardiomyocytes were cultured in 96‐well plates (4 × 10^3^ cells/well) for 48 h and treated with HG or inhibitor-5014a/inhibitor-NC. Then, 100 µL DMEM solution without FBS and 10 µL CCK‐8 reagent were added to each well and the cells were incubated at 37°C for 2 h. Absorbance (OD value) was determined at 450 nm using a microplate reader.

### 2.8 Calcein AM and ethidium homodimer III (EthD-III) staining

A 2 µM calcein AM and 4 µM EthD-III staining solution was prepared by adding 1.25 μL calcein AM and 5 μL EthD-III from the Viability/Cytotoxicity Kit for Animal Live & Dead Cells (Biotium, USA) to 2.5 mL PBS; 200 μL of the solution was added to primary cardiomyocytes in a 24-well plate. The primary cardiomyocytes were incubated at room temperature for 45 minutes, and the cells were captured using a fluorescence microscope (Nikon 80i, Japan).

### 2.9 Enzyme‐linked immunosorbent assay (ELISA)

Commercial ELISA kits were used to detect the levels of IL-1β and IL-18 in the culture media of different groups according to the manufacturer’s instructions. The OD value was measured at 450 nm using a microplate reader.

### 2.10 Gene ontology (GO) analysis and Kyoto encyclopedia of genes and genomes (KEGG) analysis

GO and KEGG analyses were employed to explore the functions and signaling pathways of differentially expressed tRFs screened using a tRF & tiRNA PCR array. GO analysis, which was conducted at http://www.geneontology.org/website, consists of three components: biological process (BP), cellular component (CC) and molecular function (MF). KEGG pathway enrichment analysis reveals dramatically enriched pathways of tRFs by analyzing a pathway-related database.

### 2.11 Protein‒protein interaction (PPI) network analysis

PPI network analysis, as carried out by the STRING database on the target genes of tRF-5014a, was used to further clarify the molecular mechanism of tRFs in HG-induced cardiomyocyte injury.

### 2.12 Statistical analysis

GraphPad Prism 8 was employed to analyze and calculate the data, which were presented as the mean ± SEM. A two-tailed Student’s t test was used to evaluate the significance of two groups, and one-way ANOVA was applied to evaluate the significance of multiple groups. The statistical analyses were performed with SPSS 25.0 software; *p* < 0.05 was considered statistically significant.

## 3 Results

### 3.1 Cardiomyocyte injury is induced by HG treatment of primary cardiomyocytes

**Figure 1:**
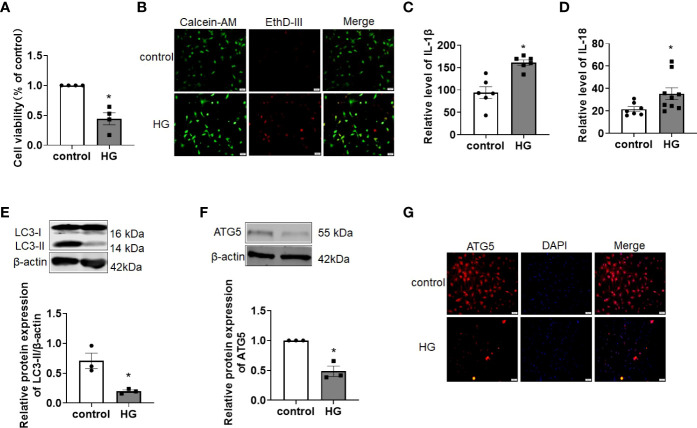
Autophagy was impaired in HG-treated primary cardiomyocytes. **(A)** The cell viability was detected by CCK-8 assay. n = 4 in each group. *P < 0.05 versus control. **(B)** The live (green) and death (red) cells were labeled with calcein-AM and EthD-III. **(C, D)** The levels of the proinflammatory cytokines IL-1β **(C)** and IL-18 **(D)** were detected by ELISA assay. n = 6-9 in each group. *P <0.05 versus control. **(E, F)** The levels of the autophagy-related proteins LC3-II **(E)** and ATG5 **(F)** were detected by western blot assay. n=3 in each group. *P < 0.05 versus control. **(G)** Immunofluorescence analysis was conducted to assess ATG5 (red) in primary cardiomyocytes of different groups. DAPI (blue) was used to stain the nucleus. HG, high glucose.

Hyperglycemia is one of the main pathogenetic factors for DCM and results in dysfunction at the cardiomyocyte cellular, structural and functional levels. Similar to previous studies, we found that HG treatment reduced the viability and induced death in primary cardiomyocytes, as detected by CCK-8 and EthD-III staining assays (**Figures 1A, B**). In addition, HG treatment resulted in higher levels of the proinflammatory cytokines IL-1β and IL-18 release (**Figures 1C, D**). As illustrated in **Figures 1E, F**, compared with the control group, the levels of the autophagy-related proteins LC3-II and ATG5 were significantly decreased after HG treatment (**Figures 1E, F**). Immunofluorescence staining confirmed the reduction in ATG5 in HG-treated primary cardiomyocytes (**Figure 1G**). These data suggest that cardiomyocyte abnormalities are induced in primary cardiomyocytes under HG conditions.

### 3.2 Differentially expressed tRFs in HG-treated primary cardiomyocytes

**Figure 2:**
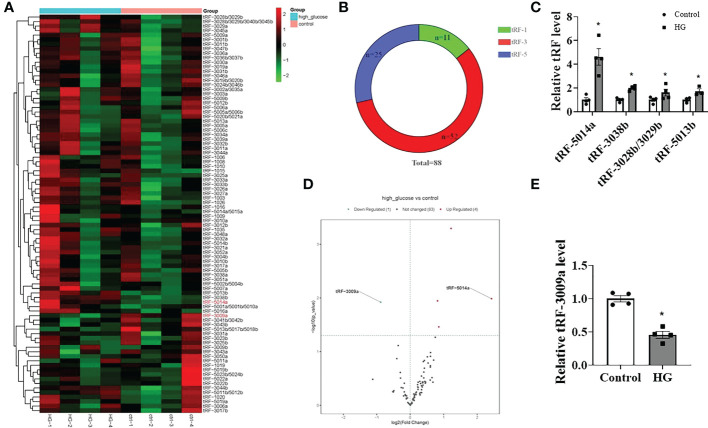
Differentially expressed tRFs were detected in HG-treated primary cardiomyocytes. **(A, B)** The tRF & tiRNA PCR array detected a total of 88 tRFs, which were classified as tRF-1 (n=11), tRF-3 (n=52) and tRF-5 (n=25). **(C–E)** Four tRFs were significantly upregulated and 1 tRF was markedly downregulated in HG-treated cardiomyocytes. n = 4 in each group. *p < 0.05 versus control.

Emerging evidence has shown that tRFs, a novel class of noncoding RNAs, contribute to multiple diseases, including neurodegenerative diseases, cancer and cardiovascular diseases. However, whether tRFs are involved in the progression of DCM associated with cardiomyocyte dysfunction is still unclear. Therefore, we first determined the changes in tRFs in HG-treated primary cardiomyocytes. As shown in **Figures 2A, B**, a total of 88 tRFs were detected by using the tRF & tiRNA PCR array and classified as tRF-1 (n=11), tRF-3 (n=52) and tRF-5 (n=25) based on the tRFdb database (http://genome.bioch.virginia.edu/trfdb/). Among them, there were significant differences in the expression of 4 upregulated tRFs (tRF-5014a, 3038b, 3028b/3029b, 5013b) and 1 downregulated tRF (tRF-3009a) in HG-treated primary cardiomyocytes, with tRF-5014a being the most upregulated (**Figures 2C–E**). These results demonstrate that HG treatment may cause dysregulation of tRFs in primary cardiomyocytes.

### 3.3 GO and KEGG pathway enrichment analysis of 4 upregulated tRFs

**Figure 3:**
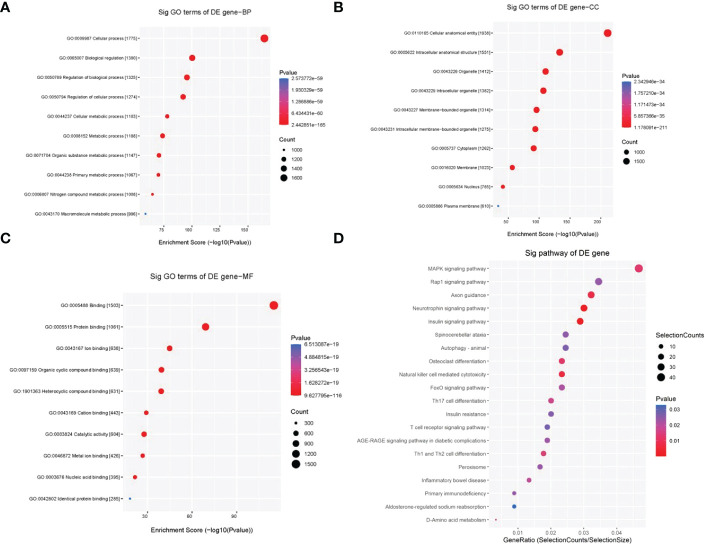
GO and KEGG pathway enrichment analyses of 4 upregulated tRFs in HG-treated primary cardiomyocytes. **(A)** BP terms mainly included cellular process, regulation of biological process, regulation of cellular process, cellular metabolic process and macromolecule metabolic process. **(B)** CC terms mainly included intracellular organelle, intracellular membrane-bounded organelle, cytoplasm, membrane-bounded organelle, membrane, nucleus and plasma membrane. **(C)** MF terms mainly included binding, protein binding, catalytic activity, nucleic acid binding and identical protein binding. **(D)** KEGG pathway enrichment analysis demonstrated the involvement of the following pathways: autophagy, AGE-RAGE signaling pathway in diabetic complications, MAPK signaling pathway, insulin signaling pathway, FoxO signaling pathway, insulin resistance and peroxisome pathways.

The above data show that HG treatment can induce both cardiomyocyte injury and tRF dysregulation. Therefore, we sought to determine whether these dysregulated tRFs are associated with HG-induced cardiomyocyte dysfunction. To address this, we predicted the potential targets of 4 upregulated tRFs based on the miRanda and TargetScan algorithms. Then, BP of GO analysis showed that the target genes of the 4 upregulated tRFs were mainly involved in cellular process, regulation of biological process, regulation of cellular process, cellular metabolic process and macromolecule metabolic process (**Figure 3A**). CC mainly referred to intracellular organelle, intracellular membrane-bounded organelle, cytoplasm, membrane-bounded organelle, membrane, nucleus and plasma membrane (**Figure 3B**). MF categories mainly included binding, protein binding, catalytic activity, nucleic acid binding and identical protein binding (**Figure 3C**). In addition, KEGG pathway enrichment analysis demonstrated the involvement of the following pathways: autophagy, AGE-RAGE signaling pathway in diabetic complications, MAPK signaling pathway, insulin signaling pathway, FoxO signaling pathway, insulin resistance and peroxisome pathways (**Figure 3D**).

### 3.4 Inhibition of tRF-5014a alleviates cardiomyocyte injury in HG-treated primary cardiomyocytes

**Figure 4:**
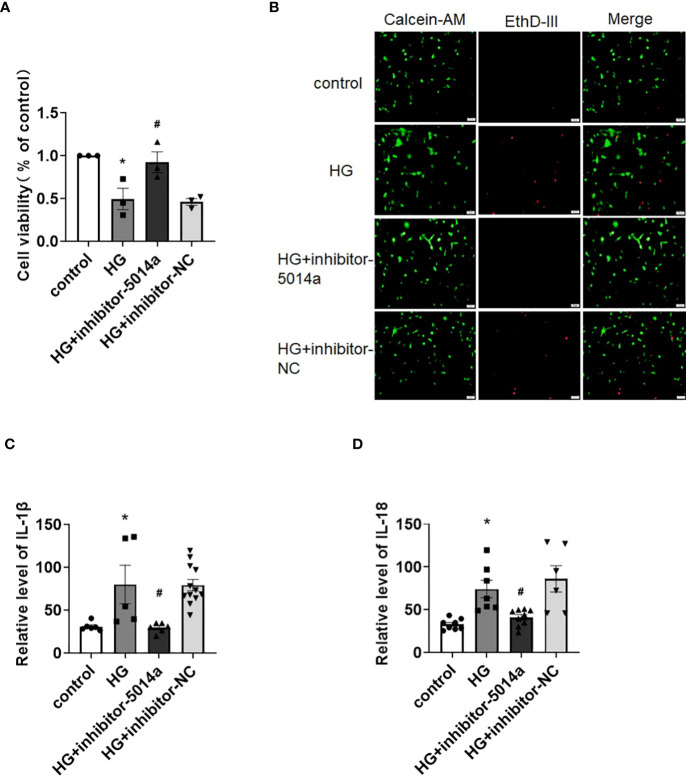
Inhibition of tRF-5014a improved cardiomyocyte injury in HG-treated primary cardiomyocytes. **(A)** The cell viability of different groups was detected by CCK-8 assay. n = 3 in each group. *P < 0.05 versus control, ^#^ *p* < 0.05 versus HG. **(B)** The live (green) and death (red) cells of different groups were labeled with calcein-AM and EthD-III. The results showed that downregulation of tRF-5014a significantly blunted the increase in cell death induced by HG treatment in primary cardiomyocytes. **(C, D)** The levels of the proinflammatory cytokines IL-1β **(C)** and IL-18 **(D)** were detected by ELISA assay. n = 5-12 in each group. *P < 0.05 versus control, ^#^ *p* < 0.05 versus HG. HG, high glucose, inhibitor-5014a: tRF-5014a inhibitor, inhibitor-NC: tRF-5014a inhibitor negative control.

As shown above, tRF-5014a was the most upregulated tRF among all tested tRFs in HG-treated cardiomyocytes. Interestingly, we observed that downregulation of tRF-5014a significantly blunted the decrease in cell viability and increase in cell death in cardiomyocytes induced by HG treatment (**Figures 4A, B**). In addition, the result of ELISA assay showed that the increases in IL-1β and IL-18 levels were blocked by transfection of tRF-5014a inhibitor (**Figures 4C, D**). These data suggested that tRF-5014a might play a critical role in cardiomyocyte injury associated with DCM.

### 3.5 tRF-5014a is involved in HG-induced cardiomyocyte dysfunction by regulating autophagy

**Figure 5:**
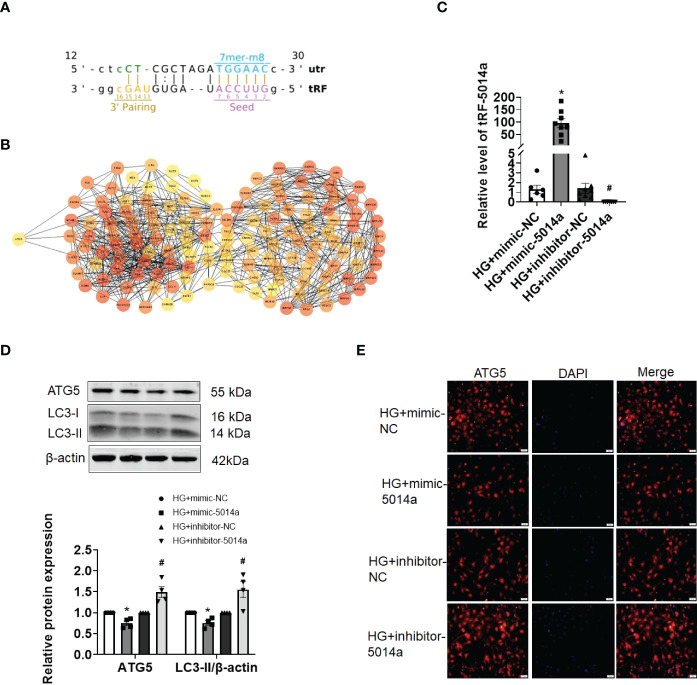
tRF-5014a regulated autophagy by targeting ATG5 in HG-treated primary cardiomyocytes. **(A)** Complementary binding site between tRF-5014a and the 3’UTR of the ATG5 gene was found based on the miRanda and TargetScan algorithms. **(B)** PPI network analysis was carried out using the STRING database on the target genes of tRF-5014a. **(C)** The tRF-5014a levels in each group were detected by real-time PCR assay after the cells were transfected with tRF-5014a mimic or inhibitor. n = 6-8 in each group. *P < 0.05 versus HG + mimic-NC, ^#^ *p* < 0.05 versus HG + inhibitor-NC. **(D)** The levels of the autophagy-related proteins LC3-II and ATG5 were detected by western blot assay. n = 4 in each group. *P < 0.05 versus HG + mimic-NC, ^#^ *p* < 0.05 versus HG + inhibitor-NC. **(E)** Immunofluorescence analysis of ATG5 (red) in each group in primary cardiomyocytes was conducted. DAPI (blue) was used to stain the nucleus. HG, high glucose; mimic-5014a, tRF-5014a mimic; inhibitor-5014a, tRF-5014a inhibitor; mimic-NC, tRF-5014a mimic negative control; inhibitor-NC, tRF-5014a inhibitor negative control.

Previous studies as well as our own have reported that autophagy inactivation results in cardiomyocyte injury, and activating autophagy is beneficial for ameliorating DCM. Next, we aimed to identify whether tRF-5014a is involved in cardiac autophagy. As shown in **Figure 5A**, there is a potential binding site between tRF-5014a and the 3’UTR of the ATG5 gene based on the miRanda and TargetScan algorithms. To further clarify the molecular mechanism of tRFs in HG-induced cardiomyocyte injury, we performed PPI network analysis, which was carried out using the STRING database, on the target genes of tRF-5014a. Many of these genes are related to the occurrence and development of diabetes and DCM. Decreased autophagy can lead to cardiac dysfunction in DCM. For example, ATG5 is a classical autophagy-related protein (**Figure 5B**). As illustrated in **Figure 5C**, tRF-5014a mimic or tRF-5014a inhibitor was successfully transfected into cardiomyocytes, as detected by real-time PCR. Importantly, we found that the level of ATG5 was decreased by tRF-5014a overexpression but increased by tRF-5014a downregulation (**Figure 5D**). Moreover, inhibition of tRF-5014a abolished the autophagy inactivation induced by HG treatment, as demonstrated by an increase in the level of LC3-II (**Figure 5D**). The change in ATG5 was also confirmed by immunofluorescence staining (**Figure 5E**). These results at least in part suggest that tRF-5014a might play a role in HG-induced autophagy dysfunction by regulating ATG5 expression during the progression of DCM.

## 4 Discussion

In the present study, we observed that cardiomyocyte injury combined with transfer RNA-derived fragment (tRF) dysfunction was induced by high glucose (HG) treatment in cardiomyocytes. We also provided evidence that these differentially expressed tRFs were primarily involved in cardiac dysfunction-related processes. Specifically, inhibition of tRF-5014a, which was the most significantly upregulated tRF, ameliorated cardiomyocyte dysfunction by regulating autophagy under HG conditions. These findings suggest that tRFs may contribute to HG-induced cardiomyocyte injury during the progression of DCM.

Diabetic cardiomyopathy (DCM) is one of diabetic complications that can result in heart failure and sudden death in diabetes mellitus patients. Nevertheless, the pathogenesis mechanisms of DCM have not yet been fully elucidated to date. It is well known that noncoding RNAs (ncRNAs) are a class of RNAs that exert regulatory effects mainly through transcriptional regulation, posttranscriptional processing or interaction with RNA-binding proteins (25). There are many types of ncRNAs, such as microRNAs (miRNAs), long noncoding RNAs (lncRNAs) and circular RNAs (circRNAs). Previous studies have shown that ncRNAs play a critical role in the pathogenesis of DCM (25, 26). Wang et al. reported that circHIPK3 is involved in inducing myocardial fibrosis by regulating miR-29b-3p, which could influence Col1a1 and Col3a1 expression during DCM (27). Our previous study also found that silencing lncRNA KCNQ1OT1 could alleviate cardiomyocyte injury and fibrosis associated with HG treatment (28). However, there are only a few studies on the relationship between tRFs and cardiovascular diseases as well as diabetic complications. For example, Shen et al. found that tRF-Gly-CCC could regulate the expression of TIMP3, thus influencing the occurrence and development of myocardial hypertrophy (29). Wang et al. identified differentially expressed tRFs in atherosclerosis (30). The data from Han’s group showed that by regulating the FoxO signaling pathway, dysregulation of tRF-Tyr-GTA-029, tRF-Thr-TGT-039 and tRF-Gln-CTG-043 contributes to the occurrence of diabetic cataracts in rats (31). However, whether and how tRFs contribute to cardiomyocyte dysfunction during DCM are still unclear to date.

In the present study, we observed that there were 4 significantly upregulated (tRF-5014a, 3038b, 3028b/3029b, 5013b) and 1 significantly downregulated tRF (tRF-3009a) in HG-treated primary cardiomyocytes, of which tRF-5014a was the most significantly upregulated. Growing evidence has shown that some tRFs function similarly to miRNAs and regulate protein expression based on sequences complementary to the 3’UTR of target genes which can be predicted using miRanda and TargetScan prediction tools (32–35). Until now, there have been no usable databases or unified algorithms for tRFs target prediction. Therefore, miRanda and TargetScan prediction algorithms were also used in our present manuscript. Herein, we found that these potential target genes of 4 significantly upregulated tRFs were enriched in cardiac dysfunction-related processes, such as autophagy, AGE-RAGE signaling pathway in diabetic complications, MAPK signaling pathway, insulin signaling pathway, FoxO signaling pathway, insulin resistance and peroxisome pathways. In addition, we proved that downregulation of tRF-5014a in HG-treated cardiomyocytes significantly abolished the decrease in cell viability and increase in cell death as well as IL-1β and IL-18 levels, suggesting that tRF-5014a might play a critical role in cardiomyocyte injury associated with DCM.

To identify the potential molecular mechanism of tRF-5014a in cardiomyocyte dysfunction, we used miRanda and TargetScan prediction algorithms to search for potential targets of tRF-5014a and found that autophagy-related protein ATG5 might be one of the targets of tRF-5014a. Autophagy is critical for maintaining intracellular homeostasis, mainly through elimination of damaged organelles and intracellular contents (36). Studies have shown that impaired autophagy can lead to abnormal cardiac function and eventually DCM (37). Moreover, as we know, decreased autophagy can also induce an increase in proinflammatory cytokines, such as IL-1β and IL-18 (38). The above evidence suggests that activating autophagy might be a beneficial therapy for ameliorating DCM by regulating ATG5 expression. Interestingly, we also revealed that tRF-5014a could regulate the expression of ATG5 and inhibition of tRF-5014a abolished autophagy inactivation under HG conditions. These results at least in part suggest that tRF-5014a might play a role in HG-induced autophagy dysfunction by regulating ATG5 expression during the progression of DCM. There are some limitations in this study, such as the lack of animal experiments, which will be addressed in the future.

In conclusion, we for the first time observed that there are some differentially expressed tRFs in HG-treated primary cardiomyocytes. In addition, we found that tRF-5014a, the most obviously differentially expressed tRF, contributes to inducing cardiomyocyte dysfunction by regulating ATG5 expression under HG conditions, suggesting that targeting tRFs might be a beneficial therapy for ameliorating DCM.

## Data availability statement

The datasets presented in this study can be found in online repositories. The names of the repository/repositories and accession number(s) can be found in the article/supplementary material.

## Author contributions

YZ and RW performed the experiments and wrote the manuscript. QQ revised the manuscript and prepared the figures. JY performed the bioinformatics analysis. HC and LW put forward the idea and revised the manuscript. All authors contributed to the article and approved the submitted version.
